# PSYCHOACOUSTICS: a comprehensive MATLAB toolbox for auditory testing

**DOI:** 10.3389/fpsyg.2014.00712

**Published:** 2014-07-21

**Authors:** Alessandro Soranzo, Massimo Grassi

**Affiliations:** ^1^Psychology, Faculty of Development and Society, Sheffield Hallam UniversitySheffield, UK; ^2^Dipartimento di Psicologia Generale, Università di PadovaPadova, Italy

**Keywords:** auditory perception, psychoacoustics, matlab toolbox, staircase, pest, maximum likelihood estimation

## Abstract

PSYCHOACOUSTICS is a new MATLAB toolbox which implements three classic adaptive procedures for auditory threshold estimation. The first includes those of the Staircase family (method of limits, simple up-down and transformed up-down); the second is the Parameter Estimation by Sequential Testing (PEST); and the third is the Maximum Likelihood Procedure (MLP). The toolbox comes with more than twenty built-in experiments each provided with the recommended (default) parameters. However, if desired, these parameters can be modified through an intuitive and user friendly graphical interface and stored for future use (no programming skills are required). Finally, PSYCHOACOUSTICS is very flexible as it comes with several signal generators and can be easily extended for any experiment.

PSYCHOACOUSTICS is a MATLAB toolbox for auditory threshold estimation. The toolbox improves and extends the Maximum Likelihood Procedure (MLP) toolbox advanced by Grassi and Soranzo (2009). Since its publication, the MLP toolbox has been extensively downloaded and has been used by both academics for teaching and research and by non-academics to test the auditory performance of their patients before and after clinical interventions (for example, Marx, 2013 utilized it to test the acoustic improvements of patients which have received cochlear implant) or to assess age-related auditory abilities (Grassi and Borella, 2013). However, MLP implements just a single adaptive procedure, and so it cannot satisfy the entire acoustic community. Hairston and Maldjian (2009), on the other hand, developed an E-Prime routine to run the Adaptive Staircase procedure. But, again, this routine implements just one adaptive procedure. Another procedure which is largely used by psychoacousticians is the Parameter Estimation by Sequential Testing (PEST). This has been implemented in Palamedes, a free MATLAB toolbox which includes functions to analyse psychophysical experiments. However, the procedure comes with no graphical interface and requires some programming skills. In sum, there are no easy to use toolboxes which implement the three most used adaptive procedures at once.

PSYCHOACOUSTICS is a new toolbox that has been developed specifically to fill this gap. It has been developed to work with MATLAB 7.0 or higher; it works with any operative system; it does not require any additional MATLAB toolboxes; and it is equipped with a user friendly and intuitive graphical interface; so, no programming skills are required. The toolbox includes the following methods:

- The Staircase—and its main variants (method of limits Fechner, 1889; Fechner, simple up-down von Békésy, 1947; transformed up-down Levitt, 1971);
- the PEST (Taylor and Creelman, 1967);
- the Maximum Likelihood (hereafter referred to as MLP Pentland, 1980; Green, 1990, 1993; Shen and Richards, 2012).

In addition, the PSYCHOACOUSTICS toolbox includes many pre-programmed experiments that, with one exception specified below, can be conducted with any of the adaptive procedures included in the toolbox. The experiments included in the toolbox are (i) the most classic psychoacoustic experiments, allowing the user to replicate established experiments or to adapt them to specific needs; (ii) experiments that, so far, have been run with non-adaptive procedures only, allowing the user to conduct the same experiments with adaptive procedures; and (iii) completely new experiments, providing the user with examples of custom usage of the toolbox and to investigate novel psychoacoustics features.

The paper is organized in three parts: The first part outlines some of the basics concepts of psychophysics (readers familiar with psychophysical concepts may wish to skip this part); the second part sketches the theory behind the three procedure types implemented in the toolbox; and finally a detailed protocol of the toolbox is outlined together with the description of the collection of psychoacoustic experiments.

## Sensory thresholds and threshold estimation

The psychophysics founder, Fechner, individuates two types of threshold: detection and discrimination (Fechner, 1889). The *detection* threshold is the minimum detectable *level* of a stimulus in the absence of any other stimuli of the same sort (where *level* indicates the acoustical parameter that is manipulated during threshold estimation). The *detection* threshold marks the beginning of the sensation of a given stimulus. Auditory examples of detection thresholds are the minimum intensity of a tone to be just detectable in silence or the minimum intensity of a tone to be just detectable when presented together with a noise (Gescheider, 2003).

The *discrimination* threshold is the minimum detectable difference between two stimuli. For a given sensory continuum, the discrimination threshold cuts the steps into those which sensory continuum is perceptually divided (Gescheider, 2003). Acoustic examples of discrimination threshold are the minimum detectable frequency difference between two tones or the minimum detectable duration difference between two tones.

*Detection* thresholds can be estimated either via *yes/no* tasks or via multiple Alternative Forced Choice tasks (in brief *nAFC*, where *n* stands for the number of alternatives). Conversely, *discrimination* thresholds are usually estimated via *nAFC* type of tasks. In *yes/no* tasks, the subject is presented with a set of isolated stimuli differing in level which spans from below to above the expected threshold. In each trial, one stimulus is presented to the subject and s/he is asked whether the stimulus has been detected (*yes*) or not (*no*). Because in *yes/no* tasks the subject's response is self-reported these responses may be biased (Green, 1993). That is, the subject could respond *yes* even in absence of any stimulus. These biased responses are called *false alarms*. Unlike *yes/no* tasks, *nAFC* task responses are not affected by false alarms because trials have correct and incorrect responses (Gescheider, 2003). In both discrimination and detection tasks the so called *lapses of attention* can occur. They are the conditions whereby subjects give the wrong response to trials that are largely over threshold (Wichmann and Hill, 2001a,b).

**Figure 1:**
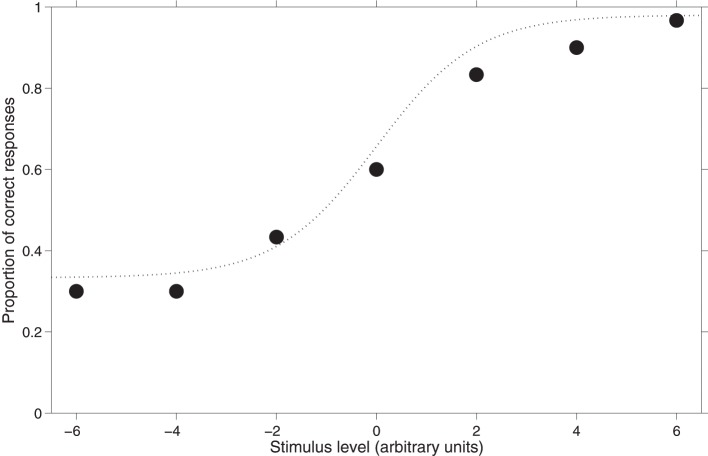
**Hypothetical results of a 3*AFC* task.** The dotted curve interpolating the subject's data points is the psychometric function.

In psychoacoustics, most of the comparisons between stimuli occur in temporal succession; for this reason *nAFC* tasks are almost invariably multiple interval tasks (*mI-nAFC*). In *mI-nAFC* tasks, in each trial the subject is presented with a set of *m* stimuli; one stimulus (variable) changes its level across trials, whereas the others (standards) are fixed. The difference between standards and variable ranges from below to above the expected detection or discrimination threshold, and subjects are asked to report which the variable stimulus was. For example, to estimate the detection threshold of a tone within noise, three noise bands may be presented in succession and only one will include the target-tone. Subjects' task would be to indicate which band contained the tone. This is a typical *3I-3AFC* task. To estimate the frequency discrimination threshold, instead, each trial may consist of two tones differing in frequency. In this case, subjects' task would be to indicate which tone has the highest pitch. This is a typical *2I-2AFC* task. In both examples, there is only one correct response and the chance level would be the reciprocal of the number of alternatives. Figure 1 shows the hypothetical results of a *3AFC* task (see Appendix).

Figure 1 shows the association between the stimulus level and the subject's performance together with a function fitting these hypothetical data. This function is referred to as the psychometric function. Independently of the task type, and of the type of threshold being measured, behavioral data are fitted with a sigmoid function such as that represented in Figure 1. Different types of psychometric functions can be adopted to fit experimental data: the logistic, the Weibull and the cumulative Gaussian are some examples. In most cases, researchers are interested in estimating just the threshold, which is a point in the psychometric function. Specifically, the threshold is an arbitrary point of the psychometric function which is defined as *p-target* (or *p*_*t*_ in formulas and “p_target” in the Graphical User Interfaces of the Psychoacoustic toolbox). Obviously, this point lies between the lower and the upper limits of the psychometric function. For the subject's threshold estimation, the procedure searches for the stimulus level eliciting the *p-target* proportion of *yes* (or correct) responses. It is debatable which *p-target* should be tracked. Treutwein (1995) suggested that the *p-target* should be the middle-point of the psychometric function. According to this suggestion, in *yes/no* tasks *p-target* should be 50% of *yes* responses, because the proportion of *yes* responses spans from 0 to 100%; in *2AFC* tasks *p-target* should be 75% of correct responses, because the proportion of correct responses spans from the chance level, 50%, to perfection, 100%; and so on. In contrast, other authors suggest selecting higher values of the *p-target* (Green, 1990; Baker and Rosen, 1998; Amitay et al., 2006). However, there is a general agreement that the *p-target* should not be less than the middle-point of the psychometric function (Green, 1990; Leek, 2001).

Thresholds can be estimated by means of two classes of procedures: non-adaptive and adaptive (Leek, 2001). In non-adaptive procedures, stimuli are pre-set before the beginning of the experiment. In these cases the stimuli span from below to above the expected threshold. One of the classic non-adaptive methods is the constant stimuli in which stimuli are presented to the subject in random order and the percentage of *yes* or correct responses is calculated for each stimulus. Thresholds are obtained by means of an interpolation procedure from the fully-sampled psychometric function resulting from the experiment.

Unlike non-adaptive procedures, adaptive procedures involve stimuli being selected in real time whilst the experiment is running. The stimulus to be presented to the subjects at each specific trial depends on the previous answers. In comparison to non-adaptive procedures, adaptive procedures maximize the ratio between the stimuli presented close to the threshold and those presented far from the threshold (Watson and Fitzhugh, 1990), hence, adaptive procedures are more efficient than non-adaptive ones. This is why they are generally preferred over non-adaptive procedures, especially when estimating just the threshold, rather than the whole psychometric function.

Adaptive procedures can be categorized as parametric (making explicit assumptions about the subject's psychometric function), and non-parametric (making no specific assumptions about the psychometric function except that it is monotonic with the stimulus magnitude). Non-parametric procedures are robust because they return veridical threshold estimations in spite of attention lapses or false alarms; however, they tend to be slow because subjects have to run many trials. In contrast, parametric procedures are faster but more vulnerable to both, attention lapses and false alarms. There is no “best” procedure, since any procedure has its pros and cons; it mostly depends on the experimenter's needs (see Leek, 2001; Marvit et al., 2003).

## Staircase, PEST, and MLP

The adaptive procedures included in the PSYCHOACOUSTICS toolbox are (i) the Staircase, (ii) the Parameter Estimation by Sequential Testing (PEST), and (iii) the Maximum Likelihood threshold estimation Procedure (MLP). These procedures have been used for decades and improved for years. Different versions of the same procedures have been proposed (e.g., Pollack, 1968; Brown, 1996; Baker and Rosen, 2001) and the next sections outline their most used variants.

### The staircase

Staircase procedures are perhaps the oldest adaptive procedures used in psychophysics. Three procedures can be distinguished within this category: the method of limits (Fechner, 1889), the simple-up down (von Békésy, 1947) and the transformed up-down (Levitt, 1971). To use any of the staircase procedures, choose “Staircase” from the dialog box that opens when running the “psychoacoustics.m” file.

#### The Method of Limits (“MethodsOfLimits” in the staircase graphical user interface)

The method of limits is commonly attributed to Fechner (1889) although this attribution has been questioned by Boring (1961). It looks for the threshold estimation on the basis of the reversal which is when the subjects change their response. Let us consider the case of the frequency discrimination threshold estimation of a 1-kHz pure tone. There will be two types of stimuli: the standard and the variable; the standard having a fixed frequency. The variable frequency will always be higher than the standard frequency by a specific Δ*f*; Δ*f* adaptively changes during the experiment. In each trial, the standard and variable are presented in a random order and the subject is asked to report the tone having the highest pitch. Every time the response is correct, Δ*f* will be reduced. In a certain trial *n*, the response will be incorrect because f will be below the sensory threshold and the subject guess is wrong. This is a reversal pattern because from a series of correct answers the procedure is now registering an incorrect one. The threshold corresponds to the average between Δ*f* and the Δ*f*_*n*−1_; that is, the average between the stimuli level before n and after the reversal (Figure 1, left graph, trial 8–9). By means of this calculation, the method of limits returns the stimulus level corresponding to the 50% of the psychometric function. In fact, the threshold calculation is made with the last level returning a correct answer (i.e., 100% of the psychometric function) and with the first level returning an incorrect answer (i.e., the 0% of the psychometric function). The method of limits can be also used to measure detection thresholds. The method of limits (as well as the simple and the transformed up-down, see below) can also be run from below; that is; the first level is below the expected threshold and it is increased in the subsequent trials; this is, however, not very common in psychoacoustics experiments.

When the initial values of both Δ*f* and Δ*f* changes are carefully selected, the method of limits results in the fastest method. However, the rapidity of the method is overtaken by the influence of chance in *nAFC* tasks and the influence of false alarms in *yes/no* tasks (Gescheider, 2003). For these reasons, this method is scarcely used in present studies.

#### The simple up-down (“SimpleUpdown” in the staircase graphical user interface)

**Figure 2:**
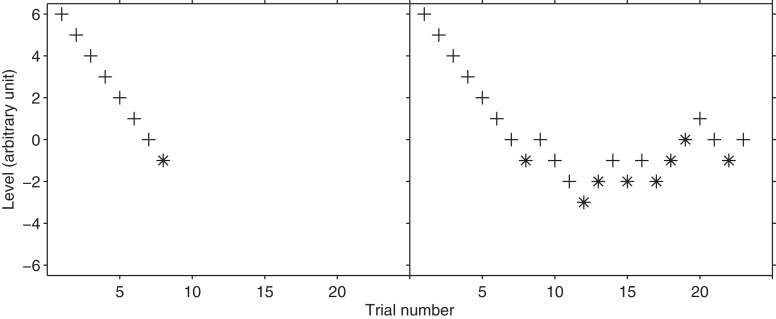
**Hypothetical threshold tracking with the method of limits (left) and with the simple up-down procedure (right).** The plus sign represents the correct responses whereas asterisk represents the incorrect responses. Note that the threshold trackings are identical up to trial n. 9. Both trackings start with a stimulus level of 6.

Some of the problems of the method of limits have been solved by the Nobel Prize research by von Békésy (1947), who advanced the variant named simple up-down. This procedure does not end at the first reversal, as it occurs in the method of limits, but it goes on until a pre-set number of reversals occur. To illustrate this procedure, let us consider the frequency discrimination example again. When the subject returns the correct choice, Δ*f* is reduced; and when the subject returns an incorrect response, the first reversal is recorded. However, as a difference from the method of limits, the experiment does not stop here but the subject is presented with at least another stimulus having an increased Δ*f*. For example, the same stimulus that was presented prior to the reversal could be presented again (right panel of Figure 2, trial 9–10). To summarize, every time the response is correct Δ*f* is reduced; whilst every time the answer is incorrect Δ*f* is increased. Like the method of limits, the simple up-down method also tracks the 50% of the psychometric function.

#### The transformed up-down

The transformed up-down advanced by Levitt (1971) can track different points of the psychometric function. This is because the up and down change of the psychometric function is attributed to the up and down change being unbalanced. In both, the method of limits and in the simple up-down, the change of the threshold tracking is balanced; that is the variable stimulus goes toward the threshold after *one* correct response and it moves away from the threshold after *one* incorrect response. For this reason the simple up-down is also defined as 1-up, 1-down procedure. In the transformed up-down, the variable stimulus moves down, toward threshold, after *two* (or more) positive responses whilst it moves up after *one* negative response.

To illustrate, let us suppose that the probability of a stimulus giving rise to a positive response is *p*. In this case, Levitt (1971) suggests moving down when the subject returns *n* positive responses (e.g., two) and to move up when the subject produces one negative response. Therefore, the probability of moving down, toward the threshold, becomes *p*^2^ whereas the probability of moving up, away from the threshold, is either *1-p* (i.e., one negative response only) or *p(1-p)*; i.e., one positive response followed by one negative response. To summarize:

$$\begin{array}{l} p^{2}=p(1-p)+(1-p)=1-p^{2}\\ \;p=\sqrt{\text{1/2}}=0.707 \end{array}$$

The 2-down 1-up (TwoDownOneUp in the Staircase Graphical User Interface) method tracks the 70.7% of the psychometric function.

There are many possible variants of this method. The most popular is the 3-down 1-up (ThreeDownOneUp in the Staircase Graphical User Interface) which tracks 79.4% of the psychometric function ($\sqrt[{3}]{1/2}$ = 0.794). It must be noted that each time the number of responses moving down is increased (e.g., from 2-down to 3-down), the length of the experiment increases because each group of “down” responses is lengthened to that of at least one trial. The psychoacoustics toolbox implements the transformed up-down up to the 4-down 1-up variant (FourDownOneUp in the Staircase Graphical User Interface).

**Figure 3:**
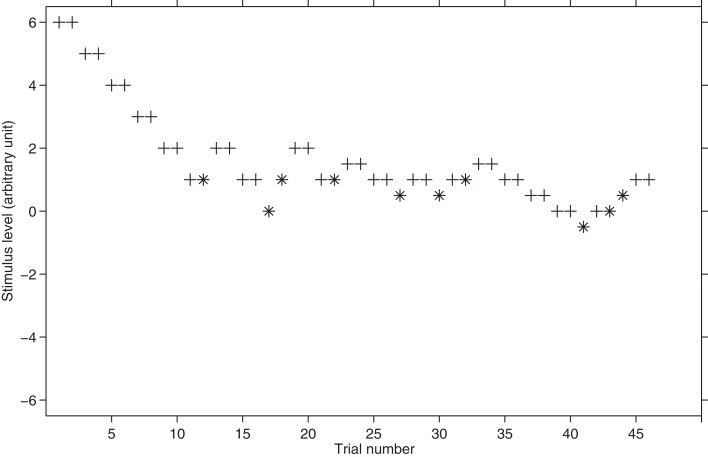
**Hypothetical threshold tracking with the transformed up-down procedure.** The plus sign represents the correct responses whereas asterisk represents the incorrect responses. The starting stimulus level is 6. The total number of reversals is 12. The first four reversals are performed with a step size of 1 and the successive eight are performed with a step size of 0.5. Note how the transformed rule lengthens the threshold tracking in comparison with the method of limits or the simple up-down procedure (see Figure 2).

The Levitt's “transformed up-down” staircase has been largely used in the last four decades. However, according to Leek (2001) the very popular 2-down 1-up is not reliable, especially when it is used in a 2*AFC* task (see also Kollmeier et al., 1988). By the same token, opting for a more robust variant (e.g., the 3-down 1-up) leads to a relatively long and arduous experiment. Figure 3 shows an example of a hypothetical threshold tracking with the transformed up-down procedure.

#### How to change the stimulus level

When using a staircase, there are two ways the stimulus level can be changed: either by addition/subtraction or by multiplication/division.

The simplest way of changing the stimulus level is to reduce/increase it by subtracting/adding a fixed amount, every time the subject returns a positive/negative response (method of limits, simple up-down) or group of responses (transformed up-down). The value of this fixed reduction/increment is called step size. For example, to estimate the absolute threshold of a sound intensity using the simple up-down method with a *yes/no* task and a step size of 1 dB; when the procedure is approaching the threshold from above, the sound intensity is reduced by 1 dB every *yes* and increased by 1 dB every *no*^1^. However, if the method of the transformed 1-up 2-down is used, the sound intensity is reduced by 1 dB every two *yeses* and incremented by 1 dB after either one *no* or after one *yes* followed by one *no*. In some cases, it may be convenient to use more than one step size: for example, a large one to approach the threshold quickly, and a small one for fine threshold estimation. In laboratory practice, a common solution is to adopt a large step size for the first 4 reversals and a smaller one in the last 8–12 reversals.

In some cases, however, the change of stimulus level by addition/subtraction is not recommended. For example, in the case of a frequency discrimination experiment, if the step size is too large the procedure can potentially move one step from a positive Δ*f* value to a negative Δ*f* value. The experimental task, “which is the highest pitch tone?,” would become ambiguous because the answer could be either the variable or the standard, depending on the Δ*f* sign. Using fixed step sizes may result in poor threshold estimation because *f* can cross the threshold too quickly. In these cases, it may be convenient to divide or multiply the step size by a certain number during the tracking (Levitt, 1971). This number is referred to as a *factor* in psychophysical papers. For example, Δ*f* could be halved after each correct response (or group of responses when using the transformed up-down) and duplicated every incorrect response (or group of responses when using the transformed up-down). In this way, *f* reaches the null value (i.e., where there is no difference between standard and variable stimuli) asymptotically only, and cannot change sign. As well as for the step size, researchers use at least two factors within a single threshold tracking: a larger factor (e.g., 2) to approach quickly the threshold and a smaller factor (e.g., $\sqrt{2}$) to stay close to the threshold in successive trials.

Whether a fixed step size or a *factor* is used to avoid lengthening the experiment, the initial value should never be too small.

#### How to calculate the threshold

In the method of limits the threshold is equal to the average between the last two levels before and after the reversal. The threshold calculation is slightly different in the simple and transformed up-down procedures. In both procedures, the threshold tracking is divided into “runs.” One run is a set of consecutive trials which includes one reversal at the end. Because each reversal is a threshold estimate, the simple up-down and the transformed up-down procedures offer several threshold estimations. Usually, the threshold is calculated by averaging the various thresholds collected during the runs. Figure 2 shows a possible threshold track arising from the simple up-down staircase. In the case shown in Figure 2, the reversals occurred at trials 8–9, 9–10, 10–11, 13–14, 16–17, 18–19, 19–20, 20–21, 22–23, and 23–24. In this case, the average of the thresholds of the last two reversals would be calculated (e.g., stimuli levels −0.5 and −1.5 in the example of Figure 2). In everyday lab-practice experimenters tend to discharge (at least) the first reversals and calculate the threshold on the successive ones. This is particularly true when the first reversals are obtained with a large factor (or step size). In conclusion, in the case of the simple and the transformed up-down procedure, the threshold is calculated by averaging either arithmetically or geometrically the various thresholds at the reversal points. Alternatively, the median can also be used.

### Parameter estimation by sequential testing (PEST)

The Parameter Estimation by Sequential Testing (PEST) procedure developed by Taylor and Creelman (1967) is the second most cited adaptive procedure in psychoacoustics, after the transformed up-down procedure. To use the PEST procedure, choose “Pest” from the dialog box that opens when running the “psychoacoustics.m” file.

This procedure is widely used within the vision community and it bases the threshold estimation on the likelihood of successive events; that is, the likelihood that the subject returns a given number of correct responses in a given number of trials. Because correct and incorrect responses are vital for PEST, this procedure cannot be used in *yes/no* tasks (this is because, for example, there is no an *AbsoluteThreshold.m* experiment in the toolbox). The algorithm of the procedure is based on the Wald sequential likelihood test (Wald, 1947). To outline the PEST procedure, let us consider again the frequency discrimination example. The experiment requires a standard stimulus and a variable stimulus whose frequencies are different by Δ*f*. The number of correct responses N(C) and the number of trials (T) are recorded during the procedure. After each trial, the Wald test defines permissible upper and lower bounds of N(C). If N(C) falls between these bounds another trial is made at the same testing level (i.e., the same Δ*f*). On the contrary, if N(C) falls outside the upper/lower bounds, *f* is considered to be too large and it has to be decreased (Taylor and Creelman, 1967).

Let us suppose that the current Δ*f* corresponds to the subject's threshold and that, in the frequency discrimination experiment, the tracked threshold is 75% of the psychometric function. In this case, by presenting Δ*f*, the expected number of correct responses E[N(C)] is *p*_*t*_ × T, where *p*_*t*_ is the *p-target*. In practice, after 100 trials, approximately 75 correct responses are expected. The following equation provides a numeric criterion to decide whether the correct responses given at Δ*f* fall within the “more or less” range, that is, whether Δ*f* is the stimulus level eliciting the 75% of correct responses:

$$N_{b}(C)=E[N(C)]\pm W$$

where *N*_*b*_(*C*) is the bounding number of events after T trials, and *W* is a constant (W constant in the PEST Graphical User Interface). When *N*_*b*_(*C*) goes outside the range set by *W* the subject has completed one run. Moreover, once *N*_*b*_(*C*) goes outside the range, the current testing level (Δ*f*) cannot be the correct threshold because the subject's performance for that particular level was either too accurate (when *N*_*b*_(*C*) > *E*[*N*(*C*)] + *W*) or too inaccurate (when *N*_*b*_(*C*) < *E*[*N*(*C*)] − *W*).

When a run is completed, the stimulus level Δ*f* changes by one step. Hence, *W* determines how rapidly and how precisely the PEST converges to the threshold. If W is small, PEST converges to a very precise threshold but in a large number of trials. If W is large, PEST converges rapidly to the threshold but the estimation may be not very accurate. Taylor and Creelman (1967) suggest setting *W* equal to 1 for a good compromise between rapidity and accuracy.

**Figure 4:**
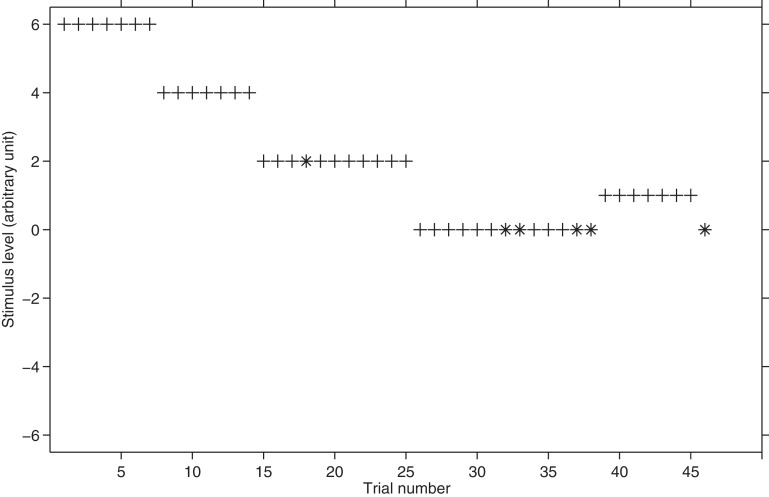
**Hypothetical threshold tracking with PEST.** The plus sign represents the correct responses whereas asterisk represents the incorrect responses. The starting stimulus level is 6. W is set to 1 and step size is initially equal to 2 and it is halved twice during the block.

Taylor and Creelman (1967) suggest following these four rules: (1) the step size has to be halved at every response reversal; (2) every time the stimulus level is changed by the same sign of the previous one, then the step size should not be changed; (3) the fourth and subsequent steps in a given direction should be double their predecessor; (4) whether a third successive step in a given direction is the same as or double the second depends on the sequence of steps leading to the most recent reversal. If the step immediately preceding that reversal resulted from a doubling, then the third step is not doubled, while if the step leading to the most recent reversal was not the result of a doubling, then this third step is the double of the second. The ideas at the basis of the rules are the following: (a) when one reversal occurs, the stimulus has to be close to the threshold and therefore it is useful to reduce the step size and stay within a range that is the midway between the levels used in the last two runs. (b) On the contrary, if PEST is moving down, toward the threshold, there is no reason to change step size unless the subject has completed several steps in a given direction. (c) In this latter case, it is more likely that the procedure is still in a region that is far from the threshold. The third rule allows rapid progression toward the threshold when the procedure is far from it. (d) The fourth rule states that to “prevent[s] rocking instability, a series of levels repeated over and over, which may happen if the third step is always doubled or always not doubled” (Taylor and Creelman, 1967; p. 784). The length of a PEST experiment depends on the step size: when the minimum step size is reached by the procedure, the experiment is concluded but no trials are actually run with that step. Figure 4 shows a hypothetical threshold tracking with PEST.

### Maximum likelihood procedure (MLP)

Among the adaptive procedures, MLP is the most recently developed. It needs many calculations so that “it turns out that the computations required to implement this technique are substantial […] so that a minimal programmable calculator is required” (Pentland, 1980; p. 377). The foundations of MLP were proposed by Pentland (1980; see also Hall, 1968) and further improvements have been advanced by Green (1993, 1995) and Gu and Green (1994). A recent update of this procedure has been proposed by Shen and Richards (2012).

To use the MLP procedure, choose “MLP” from the dialog box that opens when running the “psychoacoustics.m” file.

In MLP, the experimenter hypothesizes several psychometric functions called hypotheses. Trial by trial, the maximum likelihood algorithm estimates which hypothesis has the highest likelihood of being similar to the actual subject's psychometric function according to the subject's responses. The most likely hypothesis is assumed to contain, most likely, the threshold. MLP can track any point of the psychometric function and can be use either for *nAFC* or for *yes/no* experiments. MLP includes two independent processes: the *maximum likelihood estimation* and the *stimulus selection policy*.

#### Maximum likelihood-estimation

Before the beginning of the experiment, several psychometric functions (hypotheses) are hypothesized by the experimenter. The hypotheses share the same slope β, false alarm rate (or chance level) γ and attentional lapse rate λ, but they differ in the midpoint α so to cover the range of stimuli levels where the subject's threshold is expected to be.

After each subject's response, the likelihood of each hypothesis is calculated by means of the following function:

$$L(H_{j})=\prod_{i\;=\;1}^{n}H^{C}(x_{i}){\left[1-H(x_{i})\right]}^{W}$$

where *L(Hj)* is the likelihood of the *j*th hypothesized function, *i* is the number of trials, the exponents *C* and *W* are set to 1 and 0, respectively, when the response is *yes* (or correct) and 0 and 1, respectively, otherwise. Once the likelihood of each hypothesis has been calculated, the algorithm selects, amongst the hypothesis that one having the highest likelihood.

#### Stimulus selection policy

**Figure 5:**
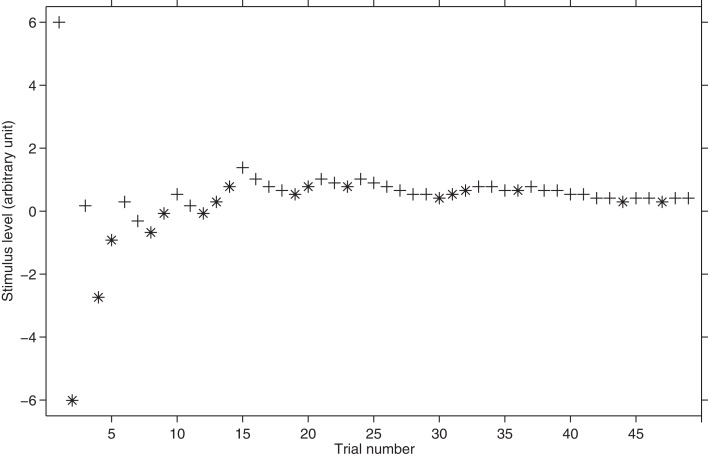
**Hypothetical threshold tracking with MLP.** The plus sign represents the correct responses whereas the asterisk represents the incorrect responses. The starting stimulus level is 6. Note how in the first trials MLP literally “jumps” between very different stimuli levels.

Once the most likely hypothesis function has been found, the next stimulus level to be presented will be the *p-target* in the function. According to Green (1990, 1993) this point, referred to as the “sweetpoint,” should optimize the estimate of the threshold; that is, it is the point at which the variance is the smallest among any other possible points included in the hypothesis function. A detailed account of this procedure can be found in Grassi and Soranzo (2009). Figure 5 shows a hypothetical threshold tracking with MLP.

#### Guidelines

*Which procedure should I use for my experiment?* As mentioned, robust threshold estimations require longer duration experiments. Of the three listed procedures, MLP is the fastest whereas transformed up-down and PEST procedure requires more time. However, MLP is less robust and threshold estimation might be affected by errors such as attention lapses. This is especially true when they occur within the first five trials of a block (Gu and Green, 1994; Grassi and Soranzo, 2009). The transformed up-down and the PEST procedures are relatively insensitive to these errors. Whilst *yes/no* experiments are relatively fast, in *nAFC* the experiment duration depends on the number of alternatives. In daily laboratory practice, *nAFC* tasks usually do not exceed four alternatives-intervals (i.e., *4I-4AFC*) otherwise the experiment duration is excessive (Schlauch and Rose, 1990). Furthermore, in the transformed up-down case, the experiment duration depends also on both the number of downs and the number of reversals. For a good compromise between duration and accuracy, the 2-down, 1-up with a *3AFC*, or a 3 down, 1-up with a *2AFC* are recommended. In doing this, the number of reversals should not exceed the number of sixteen with at least four reversals run with a large step size or factor and the remaining run with a small step size or factor. For shorter experiments the user can opt for twelve reversals, four run with a large step size or factor. In all cases, the threshold should be calculated on the reversals run with the small step size or the small factor only.

As far as PEST is concerned, Taylor and Creelman (1967) suggest setting the Wald factor to one, whilst the initial step size can be set to any value as long as it is not too large because this may result in big changes in the stimulus level from run to run, and this may disturb the subject. The same problem can arise if the upper limit of the step size is not fixed. The final step size should be chosen according to the experimenter's needs, but it has to be considered that the ratio between the initial and the final step size affects the duration of the experiment: the larger the ratio, the more reversals are needed to find the threshold.

A last recommendation is that to favor the subject's comfort, the starting level of the experiment should be sufficiently high for an easy first set of trials. However, unlike the staircase and the PEST procedures, MLP tracks the threshold by changing the stimulus level over a wide range in the first trials. Therefore, with MLP the experiment could be preceded by a short practice session or be excluded from the statistical analysis in the first block of trials.

In this section, the theoretical aspects of three procedure types implemented in the toolbox have been delineated; the remaining of this paper specifies the protocol of the toolbox and describes the built-in collection of psychoacoustic experiments.

### The psychoacoustics toolbox

PSYCHOACOUSTICS has been developed to work with MATLAB 7.0 or higher and can be downloaded from the following web site:

http://www.psy.unipd.it/~grassi/psychoacoustics.html

It works with any operative system, does not require any additional MATLAB toolboxes and does not require any programming skills^2^. The user will find the complete list of functions and experiments together with their description on the web page. The PSYCHOACOUSTICS toolbox provides an extensive number of in-built experiments; the majority of them are classic psychoacoustics experiments (e.g., frequency discrimination, intensity discrimination, etc.). Some experiments are “translations” of a set of experiments performed by Kidd et al. (2007); the user running these can compare their results with those reported in the authors' study^3^. All functions are compressed in a zip archive that the user needs to expand and copy into the MATLAB “toolbox” folder. The user also needs to add the path of the toolbox directory and its subfolders to MATLAB. All functions have a command line help function. The help can be seen by typing “help” followed by the function name at the MATLAB window.

When the toolbox is installed, the three procedures can be used as follows: Type psychoacoustics in the MATLAB prompt window to select the procedure you prefer from the dialog box (please, note that MATLAB commands are case sensitive). Each command opens a graphical interface enabling the experiment's parameters to be set and to run the experiment. The top portion of the graphical interface is similar for the three procedures and enables a subject's demographic data and the data files name to be input. Moreover, at the top of the page, the user can find two drop down menus which enable to select (and edit) The desired experiment. The bottom part of the interface enables setting the characteristics of the experiment. The labels reported in the interfaces are the same used in this paper. For example, for the staircase procedure, the step size slot enables the step size which the procedure will use during the experiment to be set (the MLP user can refer to Grassi and Soranzo, 2009, for the specific labels characterizing the MLP interface). At the bottom of the interface there are three push buttons which enable the user to quit experiment, save the parameters input by the user for later use (this should be used if the default parameters are changed) and to start the experiment. All procedures store data in two text data files. One file is labeled with the subject's name (or “untitled.txt” in the case the subject's name is missing) and contains the thresholds only. The second file is a complete record of the experiment. In each column the user will find the demographic data for each subject, the block number, the trial number, the stimulus level presented and the response. The remaining columns contain variables that are specific for each procedure. For example, in the *staircase* procedure the remaining columns are the step size and the reversal number. However, each column has a header that should help identifying its content.

#### Outline of the implemented psychoacoustic experiments

As anticipated, the toolbox comes with a number of built-in psychoacoustic experiments. The schema outlines the main features of each experiment.

#### How to respond

In all built-in experiments the subject responds by pressing the key-numbers of the computer keyboard. In nI-nAFC experiments the subject reports the temporal position of the variable stimulus. For example, in a 4AFC task, if the subject perceives the variable stimulus to be the third one, s/he must press “3”. In yes/no task, the number “1” corresponds to the “yes, I perceived/detect” answer and any other number (e.g., “0”) corresponds to “no, I don't perceive/detect”.

**Table:** 

**Experiment name**	**Description**
*AbsoluteThreshold*	Absolute threshold for a 500-ms pure tone of 1-kHz. The tone is gated on and off with two raised cosine ramps of 10-ms.
*BackwardMasking*	A 20-ms, 1-kHz pure tone (the signal) is presented immediately before (i.e., no silent gap) a band of bandpass noise of 300-ms (400–1600 Hz). All sounds are onset and offset gated by means of two raised cosine onset and offset ramps of 10-ms. The subject has to detect the tone (in yes/no task) or to tell which interval has the tone.
*ForwardMasking*	A 20-ms, 1-kHz pure tone (the signal) is presented immediately after (i.e., no silent gap) band of bandpass noise of 300-ms (400–1600 Hz). All sounds are onset and offset gated by means of two raised cosine onset and offset gates of 10-ms. The subject has to detect the tone (in yes/no task) or to tell which interval has the tone.
*SimulataneousMasking*	A 20-ms, 1-kHz sine tone (the signal) is presented in the temporal center of a band of bandpass noise of 300-ms (400–1600 Hz). All sounds are onset and offset gated by means of two raised cosine ramps of 10-ms. The subject has to detect the tone (in yes/no task) or to tell which interval has the tone.
*PitchDiscriminationPureTone*	Pitch discrimination threshold for a 250-ms, 1-kHz pure tone. The subject has to tell the highest pitch tone. Onset and offset of tones are gated on and off with two 10-ms raised cosine ramps. See Kidd et al. (2007) for possible results.
*IntensityDiscriminationPureTone*	Intensity discrimination threshold for a 1-kHz, 250-ms pure tone. The subject has to tell the loudest tone. The onset and offset of the tones are gated with two 10-ms raised cosine ramps. The standard is −30-dB attenuated in level. See Kidd et al. (2007) for possible results.
*DurationDiscriminationPureTone*	Duration discrimination for a 1-kHz, 250-ms pure tone. The subject has to tell the longest tone. The tone has raised cosine onset and offset gates of 10-ms. See Kidd et al. (2007) for possible results.
*PulseTrainDurationDiscrimination*	Pulse-train discrimination. The standard stimulus consists of six 20-ms pulses of a 1-kHz tone. These pulses are arranged in three pairs, with 40-ms of silence between members of a pair and 120 ms between pairs. The temporal structure of the variable sequence is varied by increasing the separation between members of each pair, with a corresponding decrease in the between-pair time and, thus, a constant interval between the first tones in each of the successive pairs. Thus, the first, third, and fifth tones are fixed in time, while the onsets of the second, fourth, and sixth tones are delayed by varying amounts. See Kidd et al. (2007) for possible results.
*EmbeddedTesTone*	Subjects listen for one member of a sequence of nine tones with frequencies ranging from 300 to 3000-Hz. A different, randomly selected series of nine tones is presented on each trial. The task is to detect the presence of the fifth tone in the sequence. The tone is absent in the standard. The duration of all tones except the fifth, or target tone, is 40-ms. All tones have 2.5-ms raised cosine onset and offset gates. The test is made more difficult by reducing the duration of the target tone. See Kidd et al. (2007) for possible results.
*TemporalOrderTones*	Temporal order for tones. The task is to discriminate the order in which two equal-duration pure tones of 550 and 710-Hz are presented. The duration of the two tones is varied according to listener performance. Tones are presented without a gap between them and are preceded and followed, without gaps, by 100-ms “leader” and “trailer” tones at 625-Hz. The onset and offset of the tones are gated with two 10-ms raised cosine ramps. See Kidd et al. (2007) for possible results.
*SAM_Detection_8 Hz*	Sinusoidal Amplitude Modulation (SAM) noise discrimination. A 500-ms Gaussian noise is sinusoidally amplitude modulated at 8-Hz. The depth of the modulation is expressed as 20log(m), where m is a modulation index that ranges from 0.0 (no modulation) to 1.0 (full modulation). The subject has to detect the modulation (in yes/no task) or to tell which interval has the modulated noise. Modulated and unmodulated stimuli are equated for total RMS power. Noises have two 10-ms raised cosine ramps at onset and offset. The threshold is the modulation depth (in dB). See Kidd et al. (2007) for possible results.
*SAM_Detection_20 Hz*	Sinusoidal Amplitude Modulation (SAM) noise discrimination. A 500-ms Gaussian noise is sinusoidally amplitude modulated at 20-Hz. The depth of the modulation is expressed as 20log(m), where m is a modulation index that ranges from 0.0 (no modulation) to 1.0 (full modulation). The subject has to detect the modulation (in yes/no task) or to tell which interval has the modulated noise. Modulated and unmodulated stimuli are equated for total RMS power. Noises have two 10-ms raised cosine ramps at onset and offset. The threshold is the modulation depth (in dB). See Kidd et al. (2007) for possible results.
*SAM_Detection_60 Hz*	Sinusoidal Amplitude Modulation (SAM) noise discrimination. A 500-ms Gaussian noise is sinusoidally amplitude modulated at 60-Hz. The depth of the modulation is expressed as 20log(m), where m is a modulation index that ranges from 0.0 (no modulation) to 1.0 (full modulation). The subject has to detect The modulation (in yes/no task) or to tell which interval has the modulated noise. Modulated and unmodulated stimuli are equated for total RMS power. Noises have two 10-ms raised cosine ramps at onset and offset. The threshold is the modulation depth (in dB). See Kidd et al. (2007) for possible results.
*SAM_Detection_200 Hz*	Sinusoidal Amplitude Modulation (SAM) noise discrimination. A 500-ms Gaussian noise is sinusoidally amplitude modulated at 200-Hz. The depth of the modulation is expressed as 20log(m), where m is a modulation index that ranges from 0.0 (no modulation) to 1.0 (full modulation). The subject has to detect the modulation (in yes/no task) or to tell which interval has the modulated noise. Modulated and unmodulated stimuli are equated for total RMS power. Noises have two 10-ms raised cosine ramps at onset and offset. The threshold is the modulation depth (in dB). See Kidd et al. (2007) for possible results.
*RippleNoiseDiscrimination*	Ripple noise discrimination. A 500-ms digital Gaussian noise is lowpass filtered at 3000-Hz. Sinusoidal ripples are created by adding the noise to itself with a 5-ms delay. The delayed noise is attenuated by a variable amount. The standard is always a 500-ms broadband noise with the same bandpass filtering as the “rippled” samples, but with a uniform power spectrum. Standard and variable are equalized to average RMS power. The threshold is the attenuation (in dB) of the delayed noise. See Kidd et al. (2007) for possible results.
*GapDetectionWhiteNoise*	Gap detection. A band of 750-ms gaussian noise has a gap in its temporal center. Gap duration is varied according to the listener performance. The noise has 0.5-ms cosine ramps at the beginning and end of the gap. In nI-nAFC tasks, the standard is always a 750-ms broadband noise with no gap whereas the variable contains the gap. See Kidd et al. (2007) for possible results.
*GapDiscriminationWhiteNoise*	Gap-duration discrimination. The standard is a 750-ms Gaussian noise with a silent gap of 40-ms placed at its temporal center. The variable has a variable gap duration and the length of the gap is changed as a function of the subject performance. All noises have a 0.5-ms cosine ramp at onset and offset. See Kidd et al. (2007) for possible results.
PitchDiscriminationComplexTone	Pitch discrimination threshold for a 250-ms complex tone. The tone has four harmonics (*f*_0_ = 330-Hz, mi4). The subject has to tell the highest pitch tone. Onset and offset of tones are gated on and off with two 10-ms raised cosine ramps. See Micheyl et al. (2006) for possible results.
IntensityDiscriminationComplexTone	Intensity discrimination threshold for a 250-ms complex tone. The tone has four harmonics (*f*_0_ = 330-Hz, mi4). The subject has to tell the loudest tone. The onset and offset of the tones are gated with two 10-ms raised cosine ramps. The standard is −30-dB attenuated in level.
*IntensityDiscriminationWhiteNoise*	Intensity discrimination threshold for a 250-ms white noise. The subject has to tell the loudest noise. The onset and offset of the noises are gated with two 10-ms raised cosine ramps. The standard is −30-dB attenuated in level.
*DurationDiscriminationComplexTone*	Duration discrimination for a 250-ms complex tone. The tone has four harmonics (*f*_0_ = 330-Hz, mi4). The subject has to tell the longest tone. The tone has raised cosine onset and offset gates of 10-ms.
*DurationDiscriminationWhiteNoise*	Duration discrimination for 250-ms white noise. The subject has to tell the longest noise. The noise has raised cosine onset and offset gates of 10-ms.
*ProfileAnalysis*	Profile Analysis. In this experiment the subject listens to three complex tones. Two are identical (the standards). They have five harmonics all at the same amplitude (*f*_0_ = 330-Hz, mi4). The third has a similar harmonic structure, however, the amplitude of the third harmonic component is higher producing a different timbre in comparison to the standards. The subject has to tell the odd timbre tone. The overall level of standards and variable is varied randomly from trial to trial within a range of 5-dB. Onset and offset of tones are gated on an off with two 10-ms raised cosine ramps. This experiment can be run as 3AFC only. The threshold is given in dB. Please note that the amplitude of the fixed-amplitude harmonics is −40-dB.
*MelodyMistuningDetection*	Melody mistuning detection. The major diatonic equitempered scale is played (starting do, do4 = 261.6-Hz). The sol note has a variable pitch. The subject has to tell whether the scale is in tune or out of tune (in yes/no task) or to tell the out of tune scale (in nAFC task). Notes are 500-ms complex tones of five harmonics. All tones are gated on and off with two raised cosine ramps of 10-ms. The threshold is estimated in cents. To convert the threshold in hertz: threshold = 261.6^*^2 ∧ ((700 + *t*)/1200). Where *t* is the estimated threshold in cents.

#### How to change the experiment parameters

**Figure 6:**
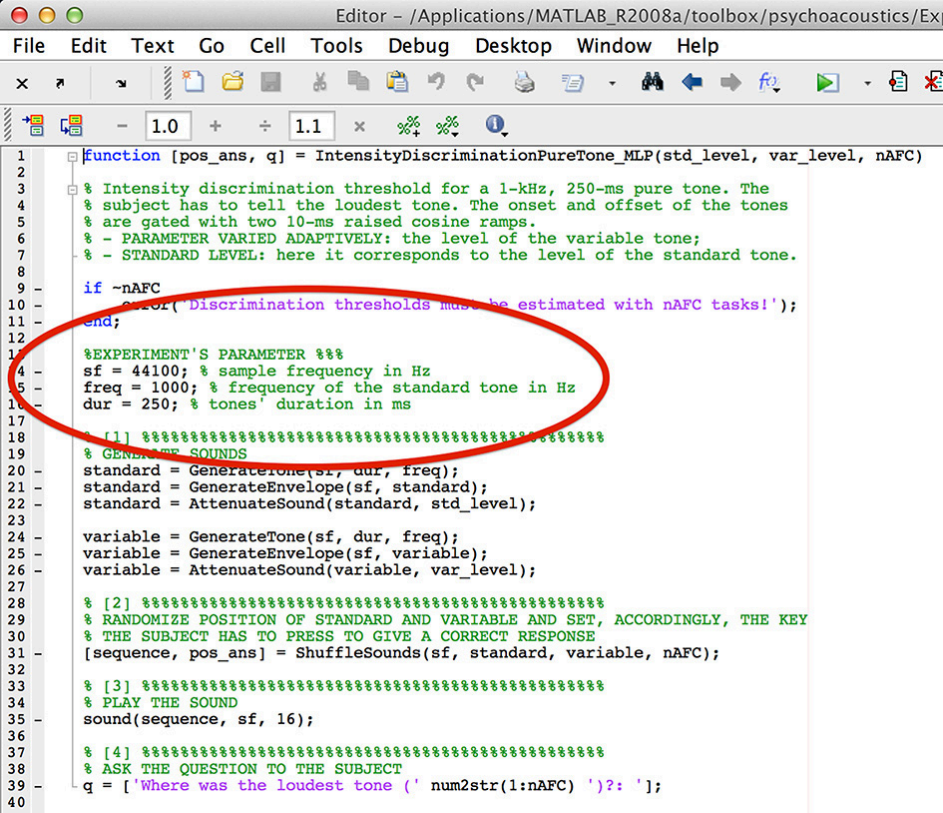
**Screenshot of the IntensityDiscriminationPureTone.m file**.

In case that the specifics of the built-in experiments do not match the experimenter's needs, they can be edited. The characteristics of the sounds are written at the beginning of the experiment.m files and can be easily manipulated. For example, in the file IntensityDiscrimination PureTone.m within the MLP folder, the frequency and the duration of the standard are fixed at 1000 and 250, respectively (Figure 6).

**Figure 7:**
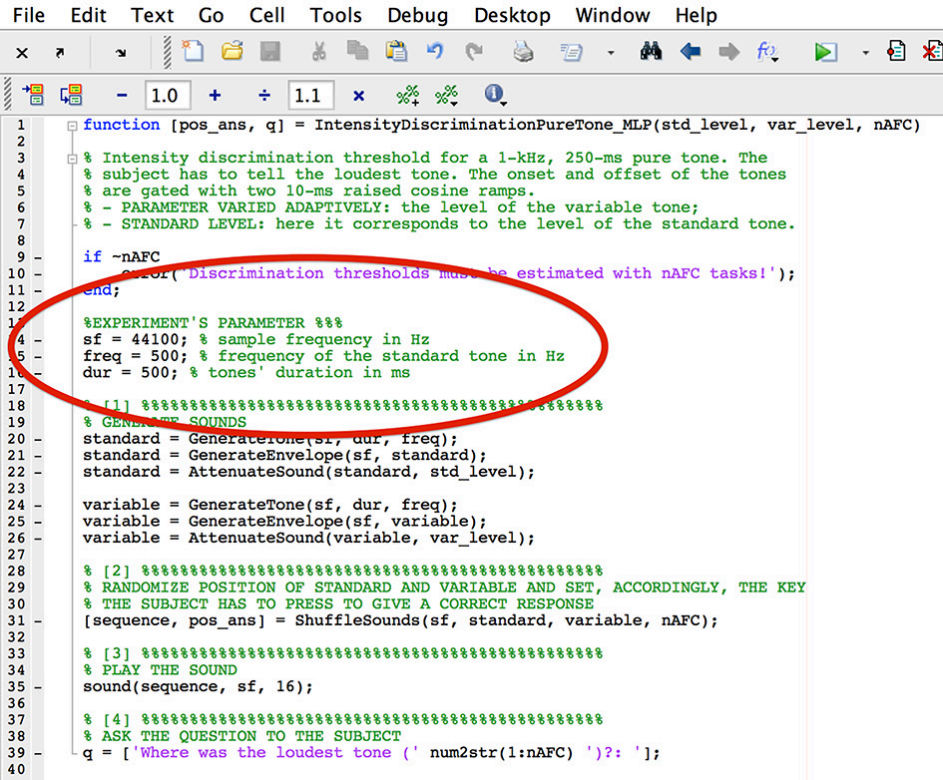
**Screenshot of the file IntensityDiscriminatioPureTone.m after the frequency and the tone duration have been changed**.

However, these values can be changed by replacing them has as shown in Figure 7. More advanced MATLAB users can write their own experiments by take as example any of the built-in experiments.

#### How to write a new experiment

The experiments in the toolbox have the same structure and they develop in four steps. It is here that sounds are generated and least one sound needs to have a variable parameter. In all built-in experiments the variable parameter is named var_level. The experiment function must also play the sound(s) to the subject and must contain a variable that tells to the toolbox which keyboard-key corresponds to a positive answer (i.e., pos_ans). In yes/no tasks this variable informs the toolbox about which key the subject has to press in order to provide a yes response. In nAFC tasks, this variable informs the toolbox which key has to be pressed to provide the correct response. Moreover, the function has to include the question to be displayed at MATLAB prompt during each trial. Finally in multiple intervals nAFC tasks, the temporal order of variable and standard should be randomized for each trial.

#### Signal generators

The psychoacoustics toolbox is provided with several signal generators and modifiers. Signal generators and modifiers are used by built-in experiment to create the sounds for the experiment. These functions can also be used to create the sounds for new experiments.

#### Toolbox calibration

Toolbox calibration is the procedure to link the sound level returned by the Psychoacoustics toolbox to the actual level produced by apparatus in use. To do this, either a sound level meter or an artificial ear is necessary. The following MATLAB commands can be used to implement and play a calibration tone (please, note that sounds level in the toolbox is in dB FS; i.e., decibels relative to the Full Scale):


sf = 44100;    % sample frequency
f = 1000;      % tone's frequency (Hz)
d = 10000;     % tone's duration (ms)
FS_level = -10; % tone's level (dB FS)
synthesize the tone
calibration_tone = GenerateTone(sf, d, f);
% set the level of the tone to "level"
calibration_tone = AttenuateSound
   (calibration_tone, FS_level);
% play the tone with the matlab "sound" command
sound(calibration_tone, sf)


The value linking the toolbox level to the actual level will be the dB SPL level (or dBA) displayed by the meter corresponding to the played calibration tone minus the FS level of the calibration tone (−10 in the example):

Linking value = db SPL level − FSlevel.

The actual threshold of a participant would be the threshold level returned by the toolbox + the linking value:

Actualthreshold = toolbox level + linkinglevel.

For example, if after playing the calibration tone the level meter displays “+60 dB SPL,” the linking level would be +70 [i.e., +60 dB SPL − (−10 dB FS)]; and if the threshold returned by the toolbox is −50 dB FS, the actual threshold would be +20 (i.e., −50 + 70).

This paper presented PSYCHOACOUSTICS, a new MATLAB toolbox for auditory threshold estimation. It is equipped with a user friendly interface and includes the adaptive psychoacoustics methods of the Staircase family, of the PEST and of the MLP. In addition, it comes with many pre-programmed experiments allowing the user to accurately replicate classical experiments by using any of the three adaptive procedures, or to adapt them for specific needs, or even to run completely new experiments. This is doable without the need of any programming skills; however, users familiar with Matlab programming may also benefit of this new toolbox by utilizing the included functions (e.g., the sound generators) as standalone functions.

### Conflict of interest statement

The authors declare that the research was conducted in the absence of any commercial or financial relationships that could be construed as a potential conflict of interest.

## Footnotes

^1^Note that because the amplitude of a sound is usually manipulated in decibels, the subtraction/addiction of a certain number of decibels results in the division/multiplication of the sound's intensity by a certain factor.

^2^Users who wish to adapt the existing experiments or who wish to develop their own experiments may find it useful to refer to the “MATLAB for Psychologists” manual (Borgo et al., 2012).

^3^Readers interested in an identical replicate of the experiments run by Kidd et al. (2007) should refer to the Test of Basic Auditory Capabilities by the same authors (Communication Disorders Technologies Inc.).

## Appendix

Figures 1–5 were obtained using simulations which hypothesized a virtual listener performing a 3*AFC* task. The responses of the virtual listener were modulated by the following psychometric function:

$$p_{c}=\gamma +(1-\lambda -\gamma )\left[\frac{1}{1+e^{\beta (\alpha -x)}}\right]$$

where *p*_*c*_ is the proportion of correct responses of the listener as a function of the level of the stimulus *x*. In the equation, γ and λ are the chance rate in the 3*AFC* task (i.e., 33%) and the lapse rate of the virtual listener (λ = 2% in all simulations), respectively. α is the psychometric function midpoint (i.e., it corresponds to the average between γ and λ, i.e., α = 65.5% in the simulated experiments) and β is the psychometric function slope (β = 1 in all simulations).

The following Table A1 reports the theoretical threshold of the virtual listeners as a function of the various *p-target*s tracked by the procedures:

**Table A1:** ***p-targets* and corresponding thresholds of the virtual listener used in the simulations**.

**Procedure**	***p-target (%)***	**Threshold (arbitrary units)**
Method of limits	50	−1.03
Simple up-down	50	−1.03
Transformed up-down	70.7	0.32
PEST	75	0.60
MLP	72.8	0.45
