# Corrigendum: PSYCHOACOUSTICS: a comprehensive MATLAB toolbox for auditory testing

**DOI:** 10.3389/fpsyg.2017.01185

**Published:** 2017-07-07

**Authors:** Alessandro Soranzo, Massimo Grassi

**Affiliations:** ^1^Psychology, Faculty of Development and Society, Sheffield Hallam UniversitySheffield, UK; ^2^Dipartimento di Psicologia Generale, Università di PadovaPadova, Italy

**Keywords:** auditory perception, psychoacoustics, matlab toolbox, staircase, pest, maximum likelihood estimation

In the “ACKNOWLEDGMENTS” there was missing information. The information is the following: this study was supported by the grant CPDA123458 (“Progetto di ricerca di Ateneo”) awarded by the University of Padova to Massimo Grassi. The authors apologize for this error and state that this does not change the scientific conclusions of the article in any way.

## Conflict of interest statement

The authors declare that the research was conducted in the absence of any commercial or financial relationships that could be construed as a potential conflict of interest.

